# Report of novel application of T-line hernia mesh in ventral hernia repair

**DOI:** 10.1016/j.ijscr.2022.106834

**Published:** 2022-02-25

**Authors:** Andrew W. Hollins, Howard Levinson

**Affiliations:** Division of Plastic, Maxillofacial, and Oral Surgery, Department of Surgery, Duke University Health System, Durham, NC, USA

**Keywords:** Ventral hernia repair, Mesh, General surgery, Plastic surgery, Innovation, Medical devices, Case report

## Abstract

**Introduction:**

Ventral hernia repair is one of the most common surgeries performed in the United States. Failure of hernia repairs can be attributed to sutures pulling through tissue or mesh (anchor point failure). T-Line Hernia Mesh is the first mesh designed to specifically prevent anchor point failure by distributing tension. This case study of two patients is the first clinical application of the novel T-Line Hernia Mesh.

**Presentation of case:**

Two separate patients presented with symptomatic ventral hernia secondary to previous laparotomy. Patient 1 is a fifty-five year-old male who underwent open ventral hernia repair with T-Line Hernia Mesh onlay placement. Patient 2 is a fifty-eight year-old female with a symptomatic ventral hernia that underwent bilateral component separation and primary hernia repair with T-Line Hernia Mesh. Both patients postoperative course was uneventful with no reported surgical site occurrences or hernia recurrence.

**Discussion:**

T-Line Hernia Mesh provides a new innovative approach to hernia surgery. This provides the first clinical outcomes. No complications were observed. In addition, this manuscript also demonstrates the surgical technique for the first time.

**Conclusion:**

This cases and technical description provides the initial report for a new designed T-Line Hernia Mesh that could result in a paradigm shift in hernia surgery concepts.

## Highlights

• T-Line Hernia Mesh® is an innovative unique design with mesh extensions that provide 15x the surface area and 275% stronger repair to overcome hernia recurrence.
• This is the first clinical study that demonstrates open ventral hernia repair with the T-Line Hernia Mesh with onlay repair. No surgical site occurrences were observed.
• T-Line Hernia Mesh design and clinical outcomes advance may advance current paradigms in hernia repair surgery mesh fixation and tools

## 1 Introduction

Ventral hernia repair (VHR) is one of the most common surgeries performed in the United States; however, surgical techniques, mesh selection, and mesh location placement vary widely [1], [2], [3]. Double blind randomized control trials illustrate long-term hernia recurrence rates >30% [4], resulting in over 400,000 ventral hernia repairs performed in the US annually [3]. It is believed that most VHRs fail because sutures (or tacks or screws, etc.) pull through fascia or mesh (termed anchor point failure, or as “cheese-wiring”), despite which mesh is used, where it is placed, how it is secured or in which patient [5], [6], [7], [8], [9]. Simply stated, despite our best efforts at pre-habilitation and surgical technique modification, we need a better performing mesh and anchoring or fixation approach to prevent hernia recurrence. To overcome anchor point failure, a novel hernia mesh titled, T-Line Hernia Mesh, was developed by Deep Blue Medical Advances Inc. based on fundamental mechanical engineering principles currently applied in tendon repair.

**Fig. 1:**
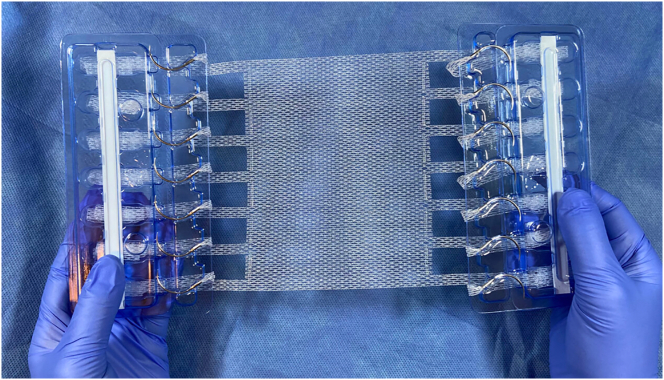
T-Line Hernia Mesh with patented suture extensions.

T-Line Hernia Mesh is a moderate weight, macroporous polypropylene mesh with mesh extensions replacing sutures that are 15 times the surface area of suture (Fig. 1). Biomechanical studies demonstrate the T-Line Hernia Mesh has 275% greater anchoring strength compared to traditional hernia mesh and the unique anchoring system allows for easy mesh application [10], [11], [12]. The design of the mesh additionally provides the possibility for increased anchor point strength over time as the extensions undergo bioincorporation. This is the first study to provide technical details of T-Line Hernia Mesh application in human ventral hernia repair and report initial clinical outcomes.

## 2 Methods

The methods of this study were approved by our institutional review board. Patients were included in our study that underwent ventral hernia repair with T-Line Hernia Mesh. Surgical dates spanned from July 2021 to September 2021. Patients were referred to physical therapy for a twelve-week postoperative hernia rehabilitation regimen. Patients were evaluated postoperatively for surgical site occurrence (SSO) or surgical site infection (SSI). SSI was diagnosed via clinical judgement by the operative surgeon. SSO included any surgical site infection, but also included seroma, wound breakdown, wound serous or purulent drainage or hernia recurrence.

Patients were evaluated for patient reported outcomes (PRO) in person or via phone after surgery. The Patient-Reported Outcomes Measurement Information System (PROMIS) Pain Intensity short form 3a and the hernia-specific quality of life (HerQLes) survey were used to assess VHR specific. The PROMIS raw scores ranged from no pain (score of three) to most painful (score of fifteen). The HerQles raw score was converted to a 100 point scale as was described in its initial implementation [13]. Higher scores are indicative of higher quality of life for the HerQles results. The SCARE 2020 guideline was utilized to meet appropriate criteria for case reports [14].

### 2.1 Surgical technique

Ventral hernia repair was done in conjunction with the general surgery service. After lysis of adhesions (LOA) was completed by the general surgery team, the plastic surgery team assessed if fascia edges were amenable to primary closure without undue tension. If the fascia could not be closed primarily, anterior component separation was performed to provide additional mobility. The fascia was closed at the midline with a size #1 Polypropylene running suture for primary closure.

**Fig. 2:**
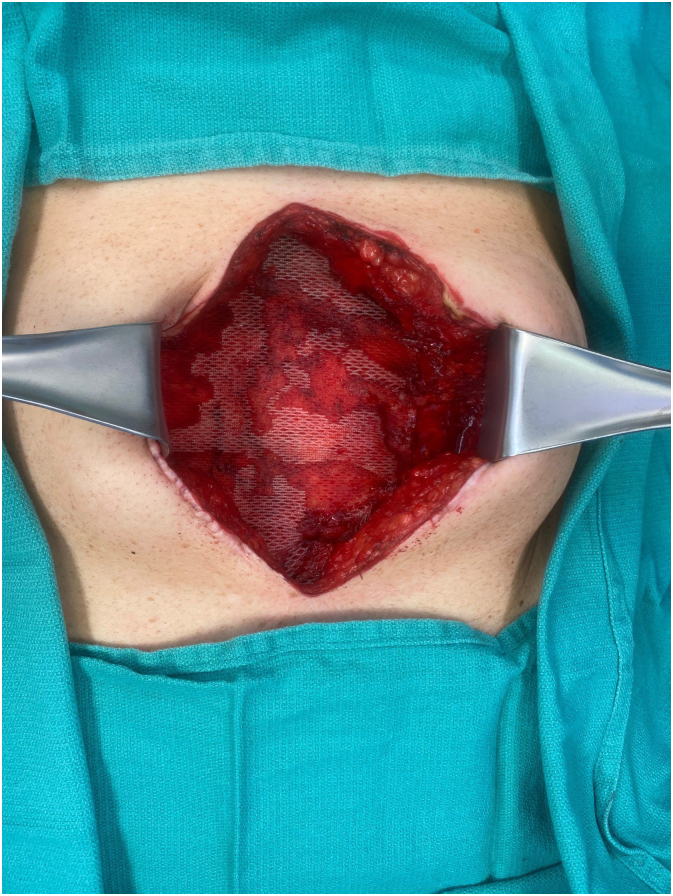
Mesh following securement to abdominal wall in patient with appropriate set tension.

Next, the T-Line Hernia Mesh was brought into the operating field. The T-Line Hernia Mesh was shaped to appropriately fit the defect with adequate overlap of the fascia edges. The tension for the mesh was set along a single side of the defect. The mesh suture was passed through the fascia at the desired point of fixation. Next, an additional pass included both the mesh and fascia for securement. Finally, a horizontal locking suture was placed including both fascia and mesh (Video 1). This process was repeated for all fixation points along the repair. The contralateral side was then secured to set the appropriate tension of the mesh and offload pressure from midline repair (Fig. 2). Mesh suture extensions were cut from the mesh and used independently to fix the mesh at the superior and inferior aspects using the same locking technique (Video 2). Skin wounds were closed in multiple layers and two suprafascial drains were placed for postoperative monitoring.

## 3 Results

Two patients were included in this series (Table 1). Both patients underwent an open hernia repair with onlay placement of T-Line Hernia Mesh® and primary closure of the fascia. The patients recovered without any SSO or noted hernia recurrence. One patient completed physical therapy postoperative regimen for abdominal core strengthening. Both patients completed postoperative patient reported outcomes.

**Table 1:** Ventral hernia repair patients.

Patient	Age gender	BMI^a^	Comorbidities	Wound status	Hernia width (cm)	Hernia length (cm)	Component separation	Mesh placement	SSO^a^	Promis 3a pain score (3–15)	HerQLes score (0–100)	Follow Up (days)
Patient 1	55 M	34.08	None	Clean	5	18	No	Onlay	No	6	50	58
Patient 2	58 F	23.14	Dysautonomia, chronic pain	Clean	5	15	Bilateral	Onlay	No	7	50	81

a SSO = Surgical Site Outcome, BMI = Body Mass Index.

### 3.1 Case 1

Patient one was a fifty-five year-old male with no significant past medical history who presented with ventral hernia following previous laparoscopic assisted donor nephrectomy in 2019. He subsequently developed a hernia in the epigastric region at the site of the hand port that caused an intermittently painful bulge. He was a former smoker with a body mass index (BMI) of 34.1. He was taken to the operating room and noted to have a 5 cm wide and 18 cm long fascial defect. The general surgery team performed a LOA and the plastic surgery team was brought to the operating room for hernia repair. The fascia was re-approximated with #1 polypropylene without the need for any component separation. An onlay repair was performed using size 10 × 30 cm (cm) T-Line Hernia Mesh. The patient was discharged on postoperative day two. His postoperative course was unremarkable. His last follow up was fifty-eight days following surgery without any noted hernia recurrence. Postoperative three-month Promis pain intensity score raw score was 6 and HerQles converted score was 50.

### 3.2 Case 2

Patient two was a fifty-eight year-old female with past medical history including chronic pain, dysautonomia, and previous ovarian cyst rupture leading to exploratory laparotomy resulting in recurrent ventral hernia. She was a former smoker with a BMI of 23.1. General surgery performed a LOA and she was noted to have a hernia defect measuring 5 cm wide and 15 cm in length. Due to the intra-operative tension noted at the midline of her fascia, a bilateral anterior component separation was performed. The fascia was then closed primarily with #1 running polypropylene. A 5 × 15 cm sized T-Line Hernia Mesh was brought onto the field and secured in place in an onlay fashion for at least 3.5 cm overlap of mesh and extensions. She was discharged from the hospital on postoperative day eight due to prolonged return of bowel function secondary to baseline dysautonomia. The patient had no noted SSO or hernia recurrence in postoperative monitoring with long term follow up of eighty-one days. She completed one month postoperative PROs with a PROMIS 3a raw pain score of 7 and HerQles converted score of 50. Her postoperative pain was unchanged from preoperatively and due to other underlying medical conditions.

## 4 Discussion

Identifying the optimal mesh for abdominal wall reconstruction can be an overwhelming task [15]. Selection of a permanent synthetic mesh may have improved long term hernia prevention without increased infectious concerns [16], [17]. The problems with fascia disruptions and traditional suture cheese-wiring in the immediate postoperative period are well described [9], [18], [19]. The T-Line Hernia Mesh is a new innovative device that allows for securement of mesh with improved strength and ease of application [10], [12]. This case series is the first demonstrated clinical application of this device.

Amato et al. has previously described a polypropylene mesh with tentacle straps for ventral hernia repair [20], [21], [22]. This model is only been demonstrated for sublay repair and require separate specific needle passer through the subcutaneous tissue. This product does not utilize a mesh suture knot for securement and instead relies on friction forces of passing through the abdominal wall. Porcine studies demonstrated that these mesh extensions are incorporated with fibrous scar as they traverse the abdominal wall. This mesh has demonstrated success in the authors findings, but no biomechanical testing for strength has been reported. The T-Line mesh offers deployment without additional instrumentation and biomechanical data supporting the strength of the repair.

The patients in this series presented with clean cases ideal for repair with synthetic mesh. The application of the novel T-Line Hernia Mesh resulted in a durable repair without any notable SSO in the immediate postoperative period. The securement with the mesh extension sutures is a fast and reliable method without the added fatigue of hand tying multiple knots. In addition, this anchor method results in a taught placement of the overlying mesh as an onlay. This provides the ideal amount of offloading pressure of the midline repair. The mesh is smooth and flat in contact with the vascularized abdominal wall. While there are many tissue planes where a mesh may be placed, onlay provides equivalent outcomes to other tissue planes in many studies [23], [24]. This eliminates any excess synthetic material that may lead to eventual nidus or prevent incorporation. Extensions can be trimmed and used multiple times and in multiple locations to anchor the mesh.

There are significant limitations in this study given its small sample size and short overall follow up. Studies have shown that hernia recurrence reporting accuracy improves with longer follow up [25]. As well, the patients selected were relatively healthy according to the ventral hernia working group classification scheme and may not reflect the broad diversity of hernia patients seen in practice [26].

The clinical application of this novel mesh provides initial findings for an innovation in hernia repair. This design could be applied to a biosynthetic or composite mesh and technique modifications are readily possible for sublay, pre-peritoneal or underlay placement. Larger clinical studies are necessary to provide additional insights for safety and performance of this mesh. This case series provides promising clinical outcomes along with technical considerations for use.

The following are the supplementary data related to this article.

Video 1 Video demonstrating securement of mesh sutures to abdominal fascia. Video 1

Video 2 Video demonstrating securement of mesh to superior aspect with individual mesh suture. Video 2

## Provenance and peer review

Not commissioned, externally peer-reviewed.

## Sources of funding

No funding was provided for this study.

## Ethical approval

The methods of this study was approved by our institutional review board.

## Consent

Written informed consent was obtained from the patient for publication of this case report and accompanying images. A copy of the written consent is available for review by the Editor-in-Chief of this journal on request.

## Research registration

N/A.

## Guarantor

Howard Levinson, MD.

## CRediT authorship contribution statement

Andrew Hollins- First author of the paper.

Howard Levinson- Senior author and surgeon who provided review and edits that significantly changed the paper.

## Declaration of competing interest

Andrew Hollins- No conflicts.

Howard Levinson- Dr. Howard Levinson is a founder of Deep Blue Medical Advances Inc. (DBMA) which has patented the device described.
